# Supplementary material for: NFE2 Truncation Mutants Protect Wild-Type NFE2 from ITCH-Dependent Degradation
Source: Int J Mol Sci. 2025 Dec 16;26(24):12112. doi: 10.3390/ijms262412112 (PMC12733253; doi:10.3390/ijms262412112)
Supplement: Supplementary file 1 [file ijms-26-12112-s001.zip › ijms-4008989-supplementary.pdf]

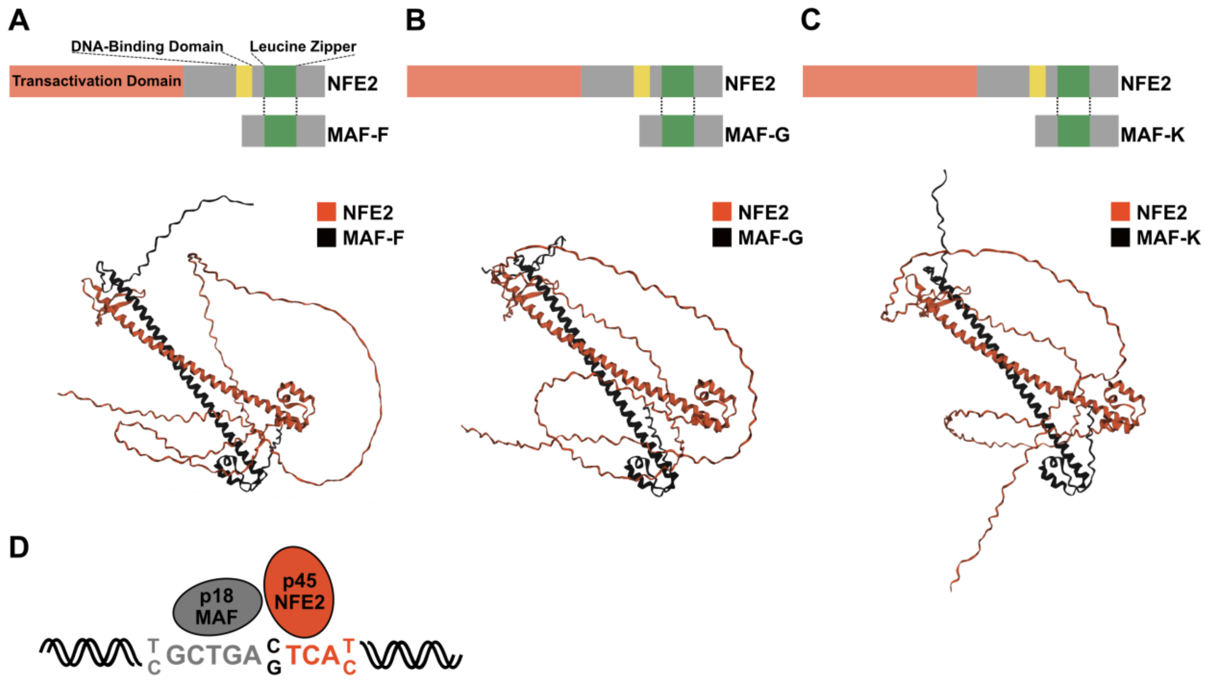

Supplemental Figure S1: Interaction between NFE2 and small MAF Proteins. (**A–C**) NFE2 forms heterodimers with the small MAF proteins (**A**) MAF-F, (**B**) MAF-G, and (**C**) MAF-K through its leucine zipper domain. Top: Schematic representation of the major domains. Bottom: AlphaFold structural predictions. Predicted Local Distance Difference Test (pLDDT) scores and predicted aligned error (PAE) plots are shown in Supplemental Figure 2. (**D**) As a heterodimer, NFE2 recognizes the AP-1-like motif (T/C)GCTGA(C/G)TCA(T/C). [40,41]

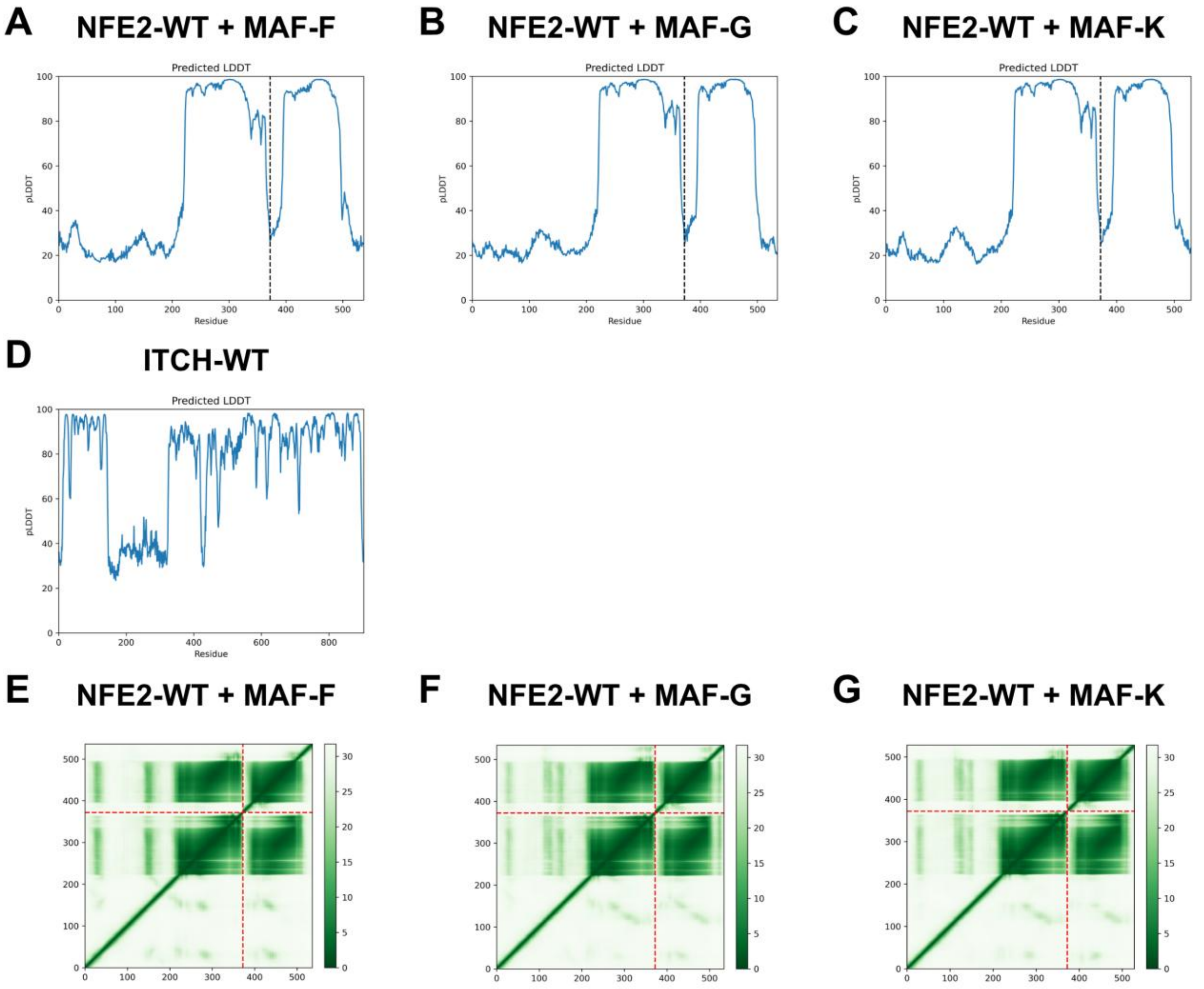

Supplemental Figure S2. Predicted Local Distance Difference Test (pLDDT) Scores and Predicted Aligned Error (PAE) Plots from AlphaFold Structural Predictions. (**A–D**) pLDDT values for (**A**) NFE2 + MAF-F, (**B**) NFE2 + MAF-G, (**C**) NFE2 + MAF-K, and (**D**) ITCH. (**E-G**) PAE plots of multimeter analyses for (**E**) NFE2 + MAF-F, (**F**) NFE2 + MAF-G, and (**G**) NFE2 + MAF-K.

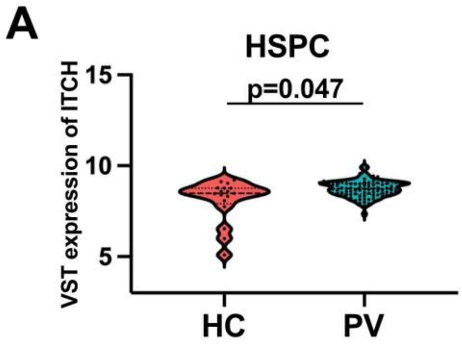

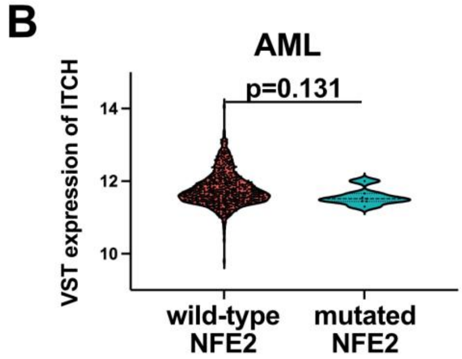

C

Samples from NFE2-mutated AML patients

Sample ID	Subject	cDNA position	Codons	Protein
BA2329D	2186	1292-1295/1833	gAGAGc/gc	p.E261Afs*3
BA2677D	2186	1292-1295/1833	gAGAGc/gc	p.E261Afs*3
BA2731D	2036	1292-1295/1833	gAGAGc/gc	p.E261Afs*3
BA3280D	2648	1171-1172/1833	gag/gGag	p.E221Gfs*7
BA3194D	2757	1321/1833	Cga/Tga	p.R271*
BA3117D	2629	1171-1172/1833	gag/gGag	p.E221Gfs*7
BA3357D	2788	1171-1172/1833	gag/gGag	p.E221Gfs*7

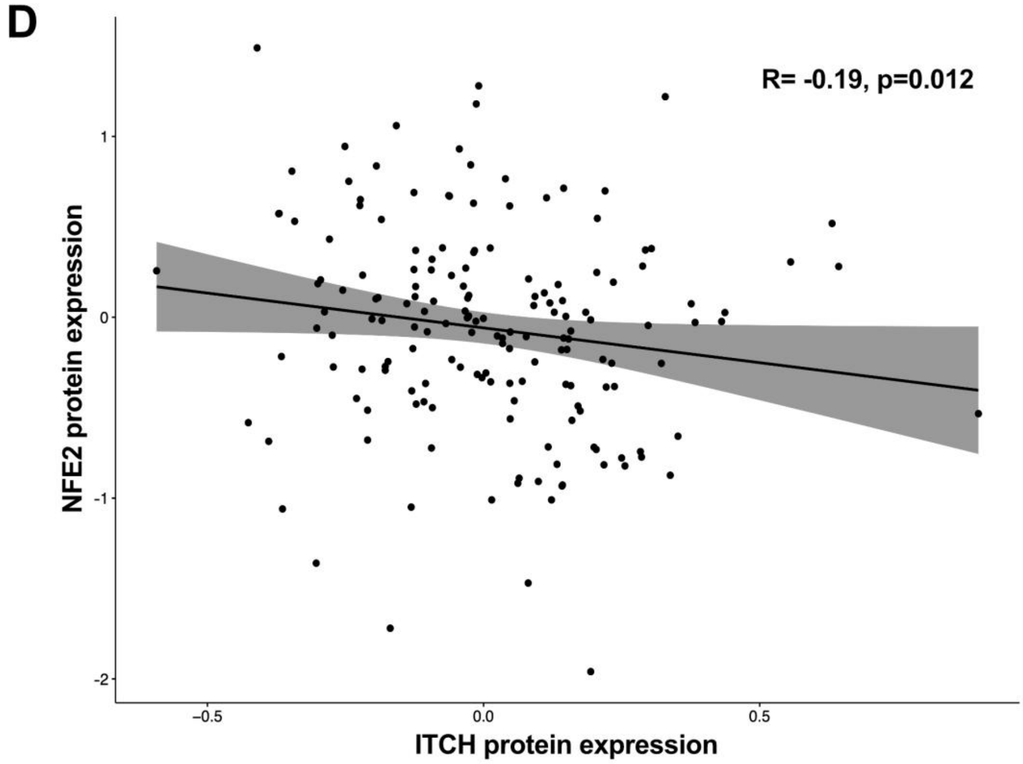

Supplemental Figure S3. ITCH Expression in MPN and AML Patients. (**A**) Violin plot showing ITCH mRNA expression in hematopoietic stem and progenitor cells (HSPCs). Data include n = 85 cell populations from patients with polycythemia vera (PV) and n = 17 cell populations from healthy controls (HC). Analysis of RNA-seq data from Tan *et al.* [28] (**B**) Violin plot showing ITCH mRNA expression in n = 664 NFE2 wild-type and n = 7 NFE2-mutated AML samples from the BeatAML cohort. [29] (**A+B**) Mean ± SEM are shown. Statistical testing was performed

using the Mann–Whitney U test. RNA-seq counts were VST-transformed. (**C**) Characterization of NFE2 mutations in the 7 NFE2-mutated RNA-seq samples derived from 6 BEAT AML patients. (**D**) Scatter plot of NFE2 and ITCH protein expression in n = 167 AML patients within the BEAT AML cohort. Association was tested using Spearman rank correlation. Mass spectrometry data were accessed from Pino *et al.* [30]

Supplemental Table S1. Types of NFE2 Mutations according to Jutzi *et al.* [5]

I A	I B	II A	II B
gain of function		loss of function	
DNA binding	No DNA binding	DNA binding	No DNA binding
Q7TfsX7	109aa	R272Q	C283R
R219Q	199aa	R273W	Δ283–284aa
K287R	215aa		R284H
	**226aa**		
	248aa		
	262aa		
	**Δ297–300aa**		

Supplemental Table S2. Transfection Mix for the $CaCl_2$ Method

DNA	Vessel	$CaCl_2$ (2.5 M)	BES buffer	ddH20
10 µg	10 cm dish	140 µl	500 µl	up to 1000 µl
2 µg	6-well plate	28 µl	100 µl	up to 200 µl

Supplemental Table S3. Transfection Mix for the PEI Method

DNA	Vessel	PEI (1 mg/mL)	IMDM
up to 5 µg	6/12-well-plate	10 µl	up to 100 µl

Supplemental Table S4. List of Plasmids

Name	Backbone	Insert
pCMV-empty	pCMV	none
pCMV-NFE2-WT	pCMV	NFE2-WT
pCMV-GFP-NFE2-WT	pCMV	GFP-NFE2-WT
pCMV-HA-NFE2-WT	pCMV	HA-NFE2-WT
pCMV-NFE2-Δ297–300	pCMV	NFE2-Δ297–300
pCMV-HA-NFE2-Δ297–300	pCMV	HA-NFE2-Δ297–300
pCMV-NFE2-226aa	pCMV	NFE2-226aa
pCMV-GFP-NFE2-226aa	pCMV	GFP-NFE2-226aa
pCMV-HA-NFE2-226aa	pCMV	HA-NFE2-226aa
pCMV-MYC-ITCH-WT	pCMV	MYC-ITCH-WT
pCMV-mCherry-MYC-ITCH-WT	pCMV	mCherry-MYC-ITCH-WT
pCMV-MYC-ITCH-C830A	pCMV	MYC-ITCH-C830A
pCMV-MafG	pCMV	MafG
pRL-TK Renilla	pRL	TK Renilla
pGL1_RBG2	pGL1	HBB_promotor

Supplemental Table S5. List of Antibodies

Target	Catalog #	Company
Anti-NFE2	HPA001914	Sigma-Aldrich – Merck, Darmstadt, Germany
Anti-MYC	Sc-40	Santa Cruz, Dallas, Texas, USA
Anti-GAPDH	G8795	Sigma-Aldrich – Merck
Anti-β-ACTIN	A5441	Sigma-Aldrich – Merck
Anti-Rabbit IgG HRP	NA934V	GE Healthcare, Chicago, Illinois, USA
Anti-Mouse IgG HRP	NA931V	GE Healthcare

Supplemental Table S6. Plasmids used for the Luciferase Assays

Plasmid	Amount
pGL1_RBG2	0.1 µg
pRL-TK Renilla	0.05 µg
pCMV-MafG	0.1 µg
pCMV-NFE2-WT	0.8 µg
pCMV-NFE2-226aa	0.8 µg
pCMV-ITCH-WT	0-3 µg
pCMV-ITCH-C830A	0-3 µg
pCMV-empty	0-3 µg

References

5. Jutzi, J.S.; Basu, T.; Pellmann, M.; Kaiser, S.; Steinemann, D.; Sanders, M.A.; Hinai, A.S.A.; Zeilemaker, A.; Bojtine Kovacs, S.; Koellerer, C.; et al. Altered NFE2 activity predisposes to leukemic transformation and myelosarcoma with AML-specific aberrations. Blood 2019, 133, 1766-1777, doi:10.1182/blood-2018-09-875047.

28. Tan, G.; Wolski, W.E.; Kummer, S.; Hofstetter, M.; Theocharides, A.P.A.; Manz, M.G.; Aebersold, R.; Meier-Abt, F. Proteomic identification of proliferation and progression markers in human polycythemia vera stem and progenitor cells. Blood Adv 2022, 6, 3480-3493, doi:10.1182/bloodadvances.2021005344.

29. Bottomly, D.; Long, N.; Schultz, A.R.; Kurtz, S.E.; Tognon, C.E.; Johnson, K.; Abel, M.; Agarwal, A.; Avaylon, S.; Benton, E.; et al. Integrative analysis of drug response and clinical outcome in acute myeloid leukemia. Cancer Cell 2022, 40, 850-864 e859, doi:10.1016/j.ccell.2022.07.002.

30. Pino, J.C.; Posso, C.; Joshi, S.K.; Nestor, M.; Moon, J.; Hansen, J.R.; Hutchinson-Bunch, C.; Gritsenko, M.A.; Weitz, K.K.; Watanabe-Smith, K.; et al. Mapping the proteogenomic landscape enables prediction of drug response in acute myeloid leukemia. Cell Rep Med 2024, 5, 101359, doi:10.1016/j.xcrm.2023.101359.

40. Talbot, D.; Grosveld, F. The 5'HS2 of the globin locus control region enhances transcription through the interaction of a multimeric complex binding at two functionally distinct NF-E2 binding sites. EMBO J 1991, 10, 1391-1398, doi:10.1002/j.1460-2075.1991.tb07659.x.

41. Mignotte, V.; Wall, L.; deBoer, E.; Grosveld, F.; Romeo, P.H. Two tissue-specific factors bind the erythroid promoter of the human porphobilinogen deaminase gene. Nucleic Acids Res 1989, 17, 37-54, doi:10.1093/nar/17.1.37.
